# Supplementary material for: General scores for accessibility and inequality measures in urban areas
Source: R Soc Open Sci. 2019 Aug 28;6(8):190979. doi: 10.1098/rsos.190979 (PMC6731692; doi:10.1098/rsos.190979)
Supplement: Supplementary Information for General scores for accessibility and inequalities in urban areas [file rsos190979supp1.pdf]

Supplementary Information for General scores for accessibility and inequalities in urban areas

Indaco Biazzo[1,*], Bernardo Monechi[2], and Vittorio Loreto[2,3,4]

[1]Politecnico di Torino, Corso Duca degli Abruzzi 24, Torino, Italy
[2]SONY Computer Science Laboratories, Paris, 6, rue Amyot, 75005, Paris, France
[3]Complexity Science Hub, Josefstädter Strasse 39, A 1080 Vienna, Austria
[4]Sapienza University of Rome, Physics Department, Piazzale Aldo Moro 2, 00185 Rome, Italy
[*]indaco.biazzo@polito.it

August 26, 2019

Abstract

This document contains the Supplementary Information for the paper "General scores for accessibility and inequalities in urban areas".

Robustness of the accessibility metrics definitions respect travel time distributions

In the definitions of the accessibility metrics, see for instance eq.(3) in the main text, we use a travel time distribution (TTD) $f(\tau)$ in order to average over time. The choice of the distribution used to average travel times is somehow arbitrary. In this section, we show that the accessibility metrics computed with differerent TTD are highly correlated with one another. Hence, different choices of TTD lead to qualitatively similar results. The first TTD considered is derived from fits of an empirical daily budget time distribution based on surveys about times spent on a bus by UK citizen [1]. From the daily budget distribution, we obtain the travel time distribution considering that on average the people perform (with a very rough approximation) two trip per day. The empirical law has been derived in [1], and it is the following:

$$f_{DBT}(t) = N * \exp(-\alpha T_{bus}/t - \beta t/T_{Bus}) \tag{1}$$

where N is a normalization constant that ensure that $\int_0^\infty f_{DBT}(t)dt = 1$. Then $\alpha = 0.2$, $\beta = 0.7$ and $T_{bus} = 67$ min are obtained by the best fit on surveys data about public transport habits. Then from the daily budget distribution eq.1 we obtain the travel time distribution computing it a $2t$, $f_{DBT}(\tau) = f_{DBT}(2t)$, where we are considering that the daily budget time is spent in two trips. This is the distribution used to compute all the quantities shown in the main text. Then we consider the travel time distribution (f_{TTD}) extracted from Oyster card journeys on bus, Tube, Docklands Light Railway and London Overground [2, 3] and a normalized flat distribution f_{1h}, between 0 and 1 hour. The distributions are shown in fig.1(panel - A). The fig.1(panel B) shown the velocity score of all points of all cities in our data-set computed with the f_{TTD} (red) and f_{1h} (green) distributions as a function of the velocity score computed with the distribution f_{DBT} used in the main text. The plot shows the high correlation between the velocity scores computed with these three different TTD. Then we check how the global quantity as the city velocity and the city sociality used to rank the city are only slightly affected by the choice of the travel time distribution. In Fig.1, panel C (panel D) it is shown the scatter plot of the city velocity (city sociality) computed with the f_{TTD} (red) and f_{1h} (green) distributions as a function of the city velocity computed with the distribution f_{DBT}. Also, in this case, the correlations between values are very high keeping in most cases the same rank order and relative distances between cities.

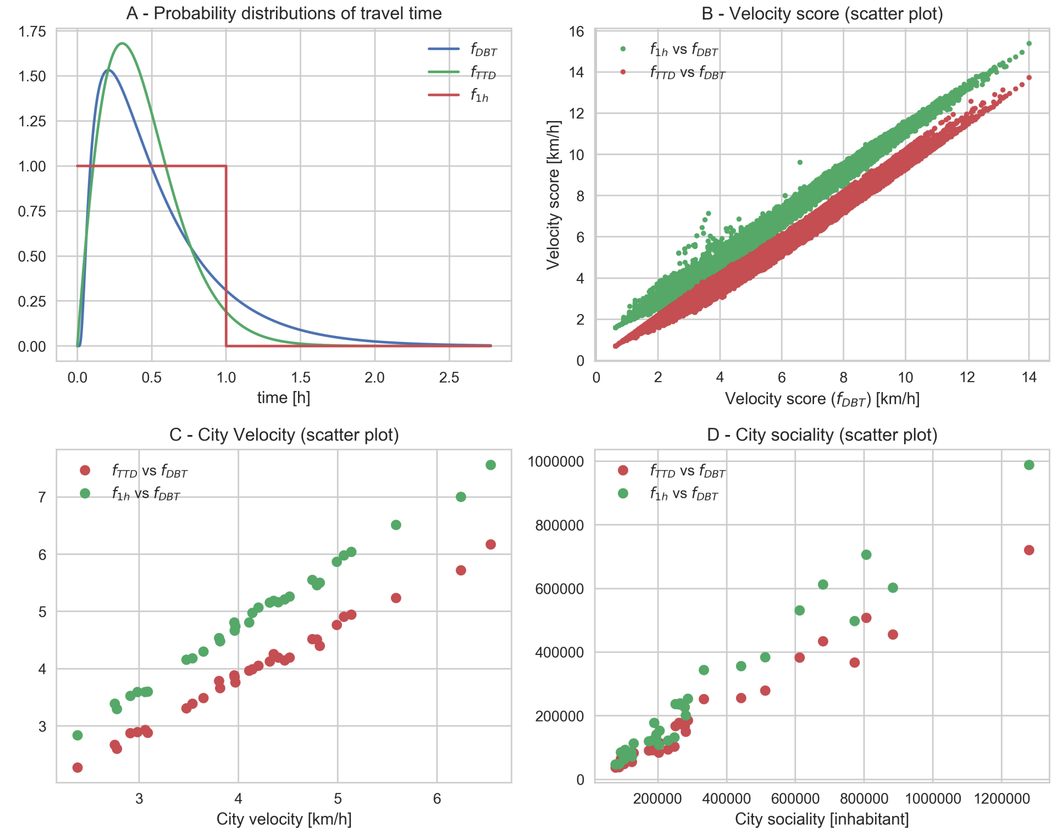

Figure 1: **Robustness of the accessibility measure definition. Panel A.** Plots of the three different travel time probability distributions. The curve $f_{DBT}(t)$ is taken from a survey on the daily use of public transports[1], the f_{TTD} is extracted from Oyster card journeys of London [2, 3], and the f_{1h} is a flat distribution between 0 and 1 hour. **Panel B.** Scatter plot of the velocity score computed in all cities with three different travel time distributions. On x-axis is the value computed with the $f_{DBT}(t)$ distribution. **Panel C(D).** Scatter plot of the city velocity(city sociality) computed with three different travel time distributions. On x-axis there is the values computed with the $f_{DBT}(t)$ distribution.

Fast and efficient routing algorithm for public transport networks

The accessibility measures computed in the present work are based on the computations of the minimum travel time between each point in the cities using public transport. Due to the fact that the number of minimum travel times to be computed is of the order 10^9 for each city, using an efficient routing algorithm is indeed mandatory. Driving times over the road network can be computed efficiently in a few milliseconds or less at the continental scale, but this is not the case of travel times with public transport [4]. This is because the approaches and speedup techniques used for road network routing algorithms fail [4] or are not so effective on public transport networks. In the last years, different approaches have emerged in literature where the most promising ones are the RAPTOR algorithm [5] and the CSA Algorithm [6]. Both these algorithms take a different approach by not looking at the graph structure of the problem. However, these algorithms have a significant limitation when considering footpaths between public transport stops in order to change means of transportation, reducing the performances in urban systems. The algorithm we used is based on the CSA algorithm, but with an essential modification that allows to use it in urban systems considering realistic footpaths to move between stops on foot and change mean of transport. We first describe the CSA algorithms and then the modified one, the Intransitive Connection Scan Algorithm (ICSA).

Connections Scan Algorithm (CSA)

A public transit network is defined by its timetable. A timetable consists of a set of stops, a set of connections and a set of footpaths. The stops are the points on the map where a traveler can enter or exit from a vehicle. A

for *all stops* s **do** $\tau[s] \leftarrow \infty$;
$\tau[s_{start}] \leftarrow t_0$;
for *all connections* c *increasing by* $t_{dep}(c)$ **do**
  if $\tau[s_{dep}(c)] \leq t_{dep}(c)$ **then**
    if $\tau[s_{arr}(c)] > t_{arr}(c)$ **then**
      $\tau[s_{arr}(c)] \leftarrow t_{arr}(c)$;
      for *all footpaths* f *from* $s_{arr}(c)$ **do**
        $\tau[f_{arr}] \leftarrow \min\{\tau[f_{arr}], \tau[s_{arr}(c)] + f_{dur}\}$;
      end
    end
  end
end

Figure 2: Connection Scan algorithm.

connection c represents a vehicle departing from the stop $s_{dep}(c)$ at time $t_{dep}(c)$ and arriving at the stop $s_{arr}(c)$ at time $t_{arr}(c)$ without intermediate halt. Movements between two stops performed on foot are called *footpaths* and are treated separately with respect to the connections. Given the timetable it is possible to compute the time needed to reach all the other stops given a starting time t_0 at the stop s_{start}. We label each stop s_i with its arrival time $\tau[s_i]$ and we set them all at starting to infinity $\tau[s_i] = \infty$ except for the starting stop $\tau[s_{start}] = t_0$ that we set to its starting time. Then we build an array containing the connections, ordered by their $t_{dep}(c)$. A connection is defined as *reachable* if the time $t_{dep}(c)$ of starting stop $s_{dep}(c)$ of the connection c is equal or larger than the time $\tau[s_{dep}(c)]$ of the stop $s_{dep}(c)$.The Connections Scan Algorithm (CSA) scans the ordered array of connections $\{c\}$, testing if each c is *reachable*. If c can be reached and if the arrival time $\tau[s_{arr}(c)]$ is larger of the arrival time $t_{arr}(c)$ of the connection, the connection is *relaxed*, meaning that the $\tau[s_{arr}(c)]$ is update to the earlier arrival time $t_{arr}(c)$. After the entire array of connections is scanned the labels *tau* contain the earliest arrival time for each stop starting from s_{start}. In the above description we do not handle footpaths. Hence, in order to consider them each time the algorithm relaxes a connection, it checks all the outgoing footpath $f[s_{arr}]$ of $s_{arr}(c)$ and updates the time of the neighbors accordingly. The algorithm requires footpaths to be *closed under transitivity* to ensure correctness. This means that if there is a footpath from stop s_a to stop s_b and a footpath from the stop s_b to the stop s_c there must be a footpath between s_a and s_c. So for every connected component of stops connected by footpaths we need all the footpaths connecting them. Since the number of footpaths grows quadratically with the number of stops, it is computationally infeasible to consider them all. In order to reduce computational time and yet considering realistic footpaths, we allowed up to 15 minutes of walk between stops. Despite with this choice all the stops of a considered city belong to the same connected component, the fact that some footpaths are not considered might lead to an overestimation of the minimum travel time between two locations due to the lack of closeness. In the next section, we describe a new version of the CSA algorithms that solves this problem. A pseudo-code of the CSA algorithm is shown in Fig. 2

Intransitive Connections Scan Algorithm (ICSA)

The variant of the CSA we propose here can correctly solve the earliest arrival time problem on public transport network considering footpaths between stops without imposing the closeness under transitivity. Let us consider the case where closeness is not enforced and all the footpaths lasting more than 15 minutes are removed. For each stop s_i consider a subsets of stops $\{s_j\}$ reachable with these footpaths. Thus, the journeys computed by the CSA algorithm could be incorrect due to missing travel time updates that should have been performed through the removed footpaths. Consider the case, we have just relaxed a connection c, i.e. the arrival time $\tau[s_i(c)]$ of the arrival stop $s_i(c)$ is updated, see Fig.3. Then the stops $\{s_j\}$, reachable by footpaths from $s_i(c)$, are checked and the arrival time $\tau[s_j^*]$ of a neighbour stop s_j^* is updated. s_j^* is also connected by footpaths to other stops $\{s_k\}$ (see Fig.3), which under closeness should have been updated through footpaths connecting them directly with s_i. However, despite they could be still updated through the footpaths starting from s_j, this does not happen because the updating of arrival time ends to the first set of neighbor stops. In worst cases, it could also happen that the remaining connections that arrive on s_j never relax, because all of them arrive after the time $\tau[s_j]$. Hence, no one of its neighbors will be updated through footpaths connecting them to s_j. However, there could be journeys passing through s_j that do not update $\tau[s_j]$ directly, but they might update some of its neighbors $\{s_k\}$ through

footpaths. The CSA algorithm is not able to consider this. The Intransitive Connections Scan algorithms (ICSA)

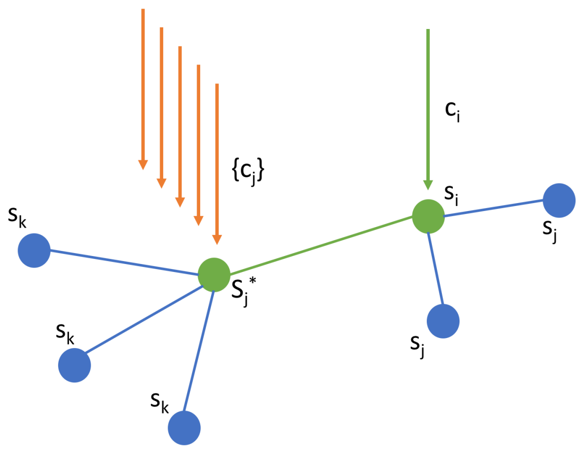

Figure 3: In this cartoon shows the updating process of the CSA algorithm and the error when the closeness by transitivity of the footpaths between stops is not considered. First a connection c arriving at the stop s_i relaxes, i.e. update the arrival time $\tau[s_i]$ of s_i. Then the arrival time $\tau[s_j^*]$ of the neighbour stop s_j^* is update through the footpath. Then other connections $\{c_j\}$ arriving to s_j^* do not relax and the neighbour $\{s_k\}$ are never update by footpaths from s_j^* causing possible errors.

overcomes this problem with a small modification of the original CSA algorithm, without increasing the running time and preserving its simplicity. The key is to consider two labels τ and τ^f for the arrival time for each stops. One represents the arrival time to the stop after the relaxation of one connection and the other due to the updating of the arrival time by footpaths. The ICSA algorithm is the same as CSA except for three modifications:

1. a connection is considered reachable if its starting time $t_{dep}(c)$ is larger or equal either to arrival time of the stops $\tau[s_{dep}(c)]$ due to connection updating or to the arrival time $\tau^f[s_{dep}(c)]$ due to the footpaths update.

2. Both the CSA and ICSA update the arrival time of the stops in two ways: by direct relaxation of the connections or by footpaths. The ICSA algorithm updates the arrival time $\tau[s]$ of the stop s when it is updated by connections, and the arrival time $\tau^f[s]$ when it is updated by footpaths.

3. After the complete scanning of the connections array, the arrival time taken for each stops s is the best arrival time between the two labels $tau[s]$, $tau^f[s]$.

A pseudo-code implementation scheme is shown in fig.4.

These modifications allow to correctly solve the problem of finding the earliest arrival time to stops, given a set of connections and footpaths connecting them, without the constraint of the closeness under transitivity of footpaths.

for *all stops* s **do** $\tau[s] \leftarrow \infty$;
for *all stops* s **do** $\tau^f[s] \leftarrow \infty$;
$\tau[s_{start}] \leftarrow t_0$;
for *all connections* c *increasing by* $t_{dep}(c)$ **do**
  if $\tau[s_{dep}(c)] \leq t_{dep}(c)$ *or* $\tau^f[s_{dep}(c)] \leq t_{dep}(c)$ **then**
    if $\tau[s_{arr}(c)] > t_{arr}(c)$ **then**
      $\tau[s_{arr}(c)] \leftarrow t_{arr}(c)$;
      for *all footpaths* f *from* $s_{arr}(c)$ **do**
        $\tau^f[f_{arr}] \leftarrow \min\{\tau^f[f_{arr}], \tau[s_{arr}(c)] + f_{dur}\}$;
      end
    end
  end
end
for *all stops* s **do** $\tau[s] \leftarrow min(\tau[s], \tau^f[s])$;

Figure 4: Intransitive Connection Scan Algorithm

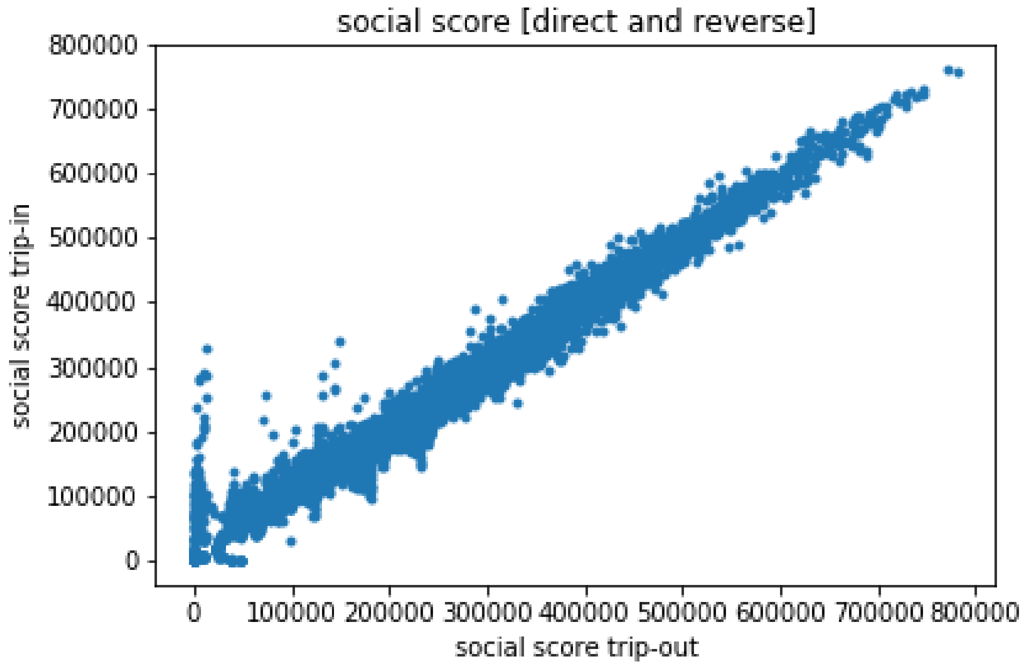

Figure 5: **Scatter plot of sociality score compute with travel time of incoming and out-coming trip.** The sociality score is based on the computation of the travel time of trips connecting the point considered to all the others. When considering the travel time of the out coming trips the sociality score represents the amount of people is possible to reach at home from the considered point. Instead when the travel time of incoming trips are taken into account the sociality score represent the amount of people can easily reach the considered points. The two quantity are very similar to each others, because, on average, the travel time trip of the out-coming and incoming trips are very similar. In the figure there is the scatter plot between the sociality score computed with incoming and outcoming trips for Rome.

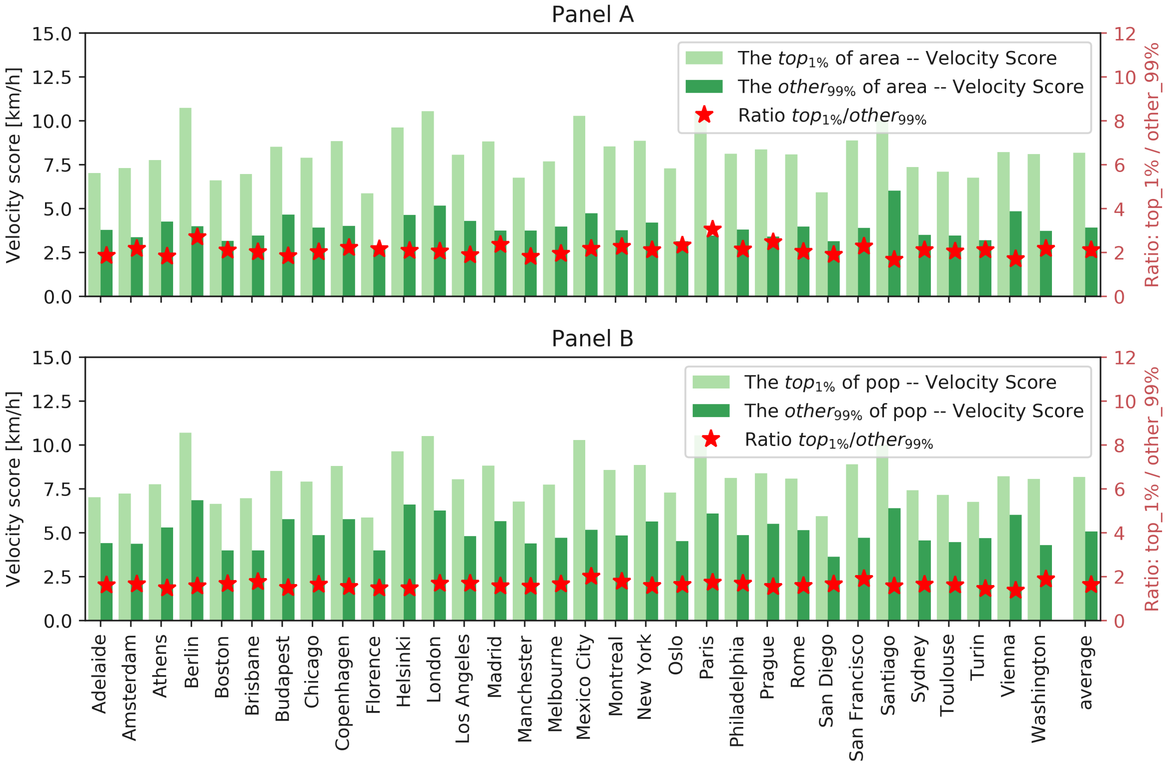

Figure 6: **Panel A**: Average values of the velocity scores among the hexagons featuring the top 1% (light green) of the values of the velocity score as compared to the remaining 99% (dark green) for all cities analyzed. The last columns report the same values averaged over all cities. Red stars mark, on the right y-axis, are the ratios between the average values of top 1% and the other 99%. **Panel B**: Average values of the velocity scores among the population with the highest top 1% (light green) of the values of the velocity score as compared to the remaining 99% (dark green) for the six selected cities. The last columns report the same values averaged over all the six cities. Red stars mark, on the right y-axis, are the ratios between the average values of top 1% and the other 99%.

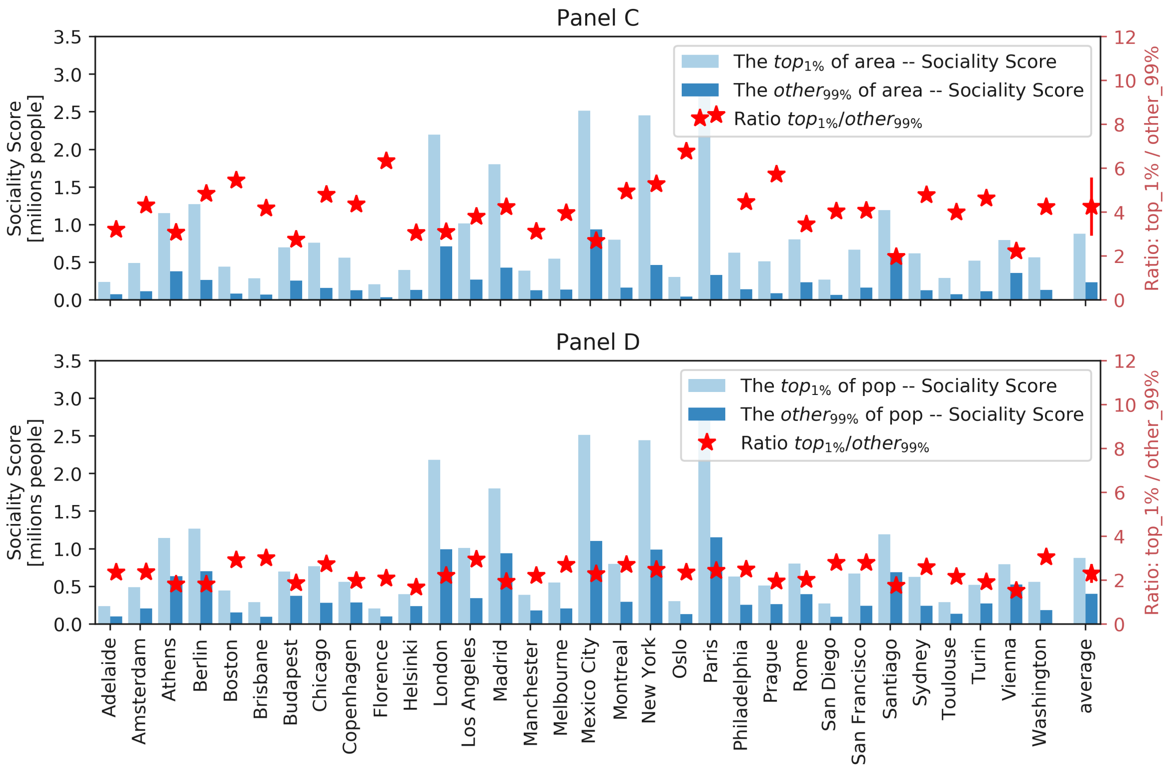

Figure 7: **Panel C**: Same as Panel A of Fig.6 for the Sociality score. **Panel D**: Same as Panel B of Fig.6 for the Sociality score.

References

[1] Kölbl R, Helbing D. Energy laws in human travel behaviour. New Journal of Physics. 2003;5(1):48.

[2] Gallotti R, Bazzani A, Rambaldi S. Understanding the variability of daily travel-time expenditures using GPS trajectory data. EPJ Data Science. 2015;4(1):18. Available from: `http://dx.doi.org/10.1140/epjds/s13688-015-0055-z`.

[3] Gallotti R, Bazzani A, Rambaldi S, Barthelemy M. A stochastic model of randomly accelerated walkers for human mobility. Nature Communications. 2016 08;7:12600. Available from: `http://dx.doi.org/10.1038/ncomms12600`.

[4] Bast H, Delling D, Goldberg A, Mller-Hannemann M, Pajor T, Sanders P, et al. Route Planning in Transportation Networks. In: Algorithm Engineering. Springer International Publishing; 2016. p. 19–80. Available from: `https://doi.org/10.1007%2F978-3-319-49487-6_2`.

[5] Delling D, Pajor T, Werneck RF. Round-based public transit routing. Transportation Science. 2014;49(3):591–604.

[6] Dibbelt J, Pajor T, Strasser B, Wagner D. Intriguingly simple and fast transit routing. In: International Symposium on Experimental Algorithms. Springer; 2013. p. 43–54.

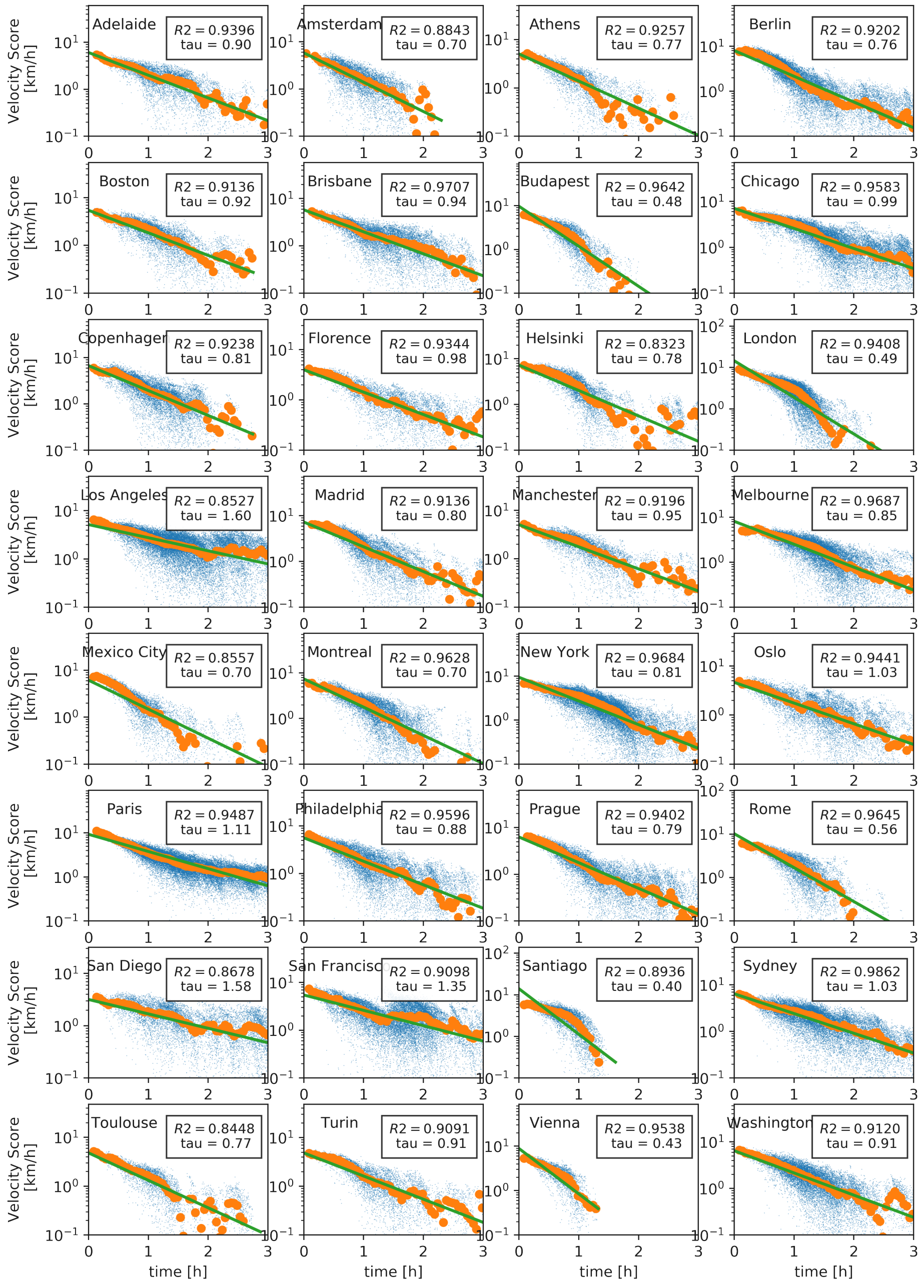

Figure 8: **Exponential decay of the velocity score with travel-times for the city center.** Velocity scores of hexagons at a given travel-time distance from the hexagon with the highest velocity score in each of the six selected city (blue dots). The orange points report a binning of the blue dots. The green line is the best fit of the data with the function 8 in the main text.

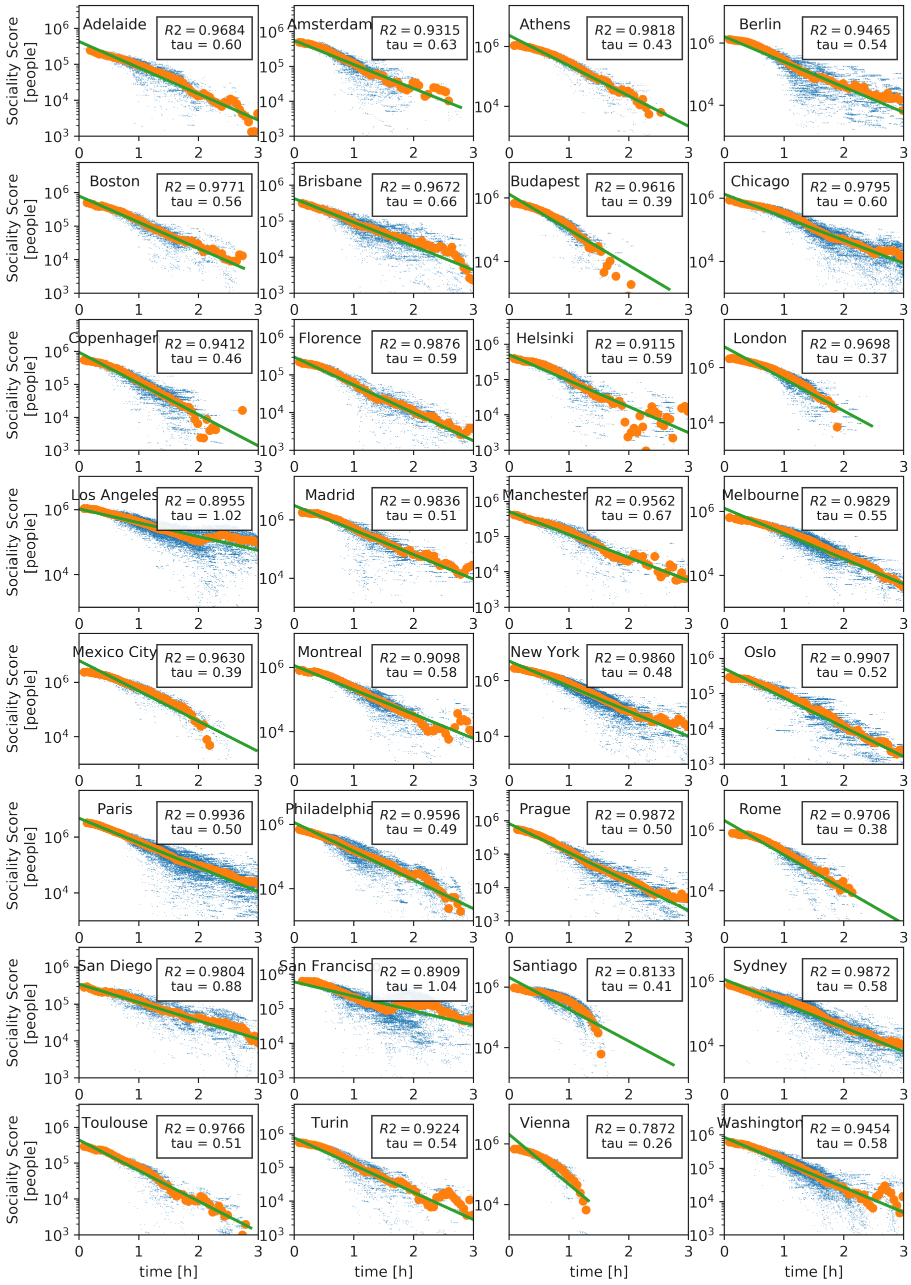

Figure 9: **Exponential decay of the sociality score with travel-times for the city center.** Sociality scores of hexagons at a given travel-time distance from the hexagon with the highest sociality score in each of the six selected city (blue dots). The orange points report a binning of the blue dots. The green line is the best fit of the data with the function 8 in the main text.
